# Preserving Shell Integrity of Microgastropods With the Nondestructive HotSHOT DNA Extraction Method

**DOI:** 10.1002/ece3.74031

**Published:** 2026-07-15

**Authors:** Nina Tombers, Cristina Vasilița, Ira Richling

**Affiliations:** ^1^ Stuttgart State Museum of Natural History Stuttgart Germany; ^2^ Vogelwarte, Zoologisches Institut Und Museum Ernst‐Moritz‐Arndt‐Universität Greifswald Greifswald Germany; ^3^ Karlsruhe Institute of Technology, Institute for Photon Science and Synchrotron Radiation (IPS) Eggenstein‐Leopoldshafen Germany; ^4^ Center for Biodiversity and Integrative Taxonomy Research, Institute of Biology, University of Hohenheim, Stuttgart Germany

**Keywords:** DNA barcoding, molecular method, Mollusca, species‐level identification, voucher

## Abstract

Gastropods rank among the most species‐rich animal classes, accounting for 5% (4–8) of all described animal species with about 1/3 of microgastropods (< 5 mm in size). DNA extraction from preserved microgastropods retracted into their shells is challenging because established methods usually damage the shell. This study tests the HotSHOT alkaline lysis method as a nondestructive protocol for small ethanol‐preserved gastropods. Using a brief lysis step at high pH and temperature, we achieved effective DNA extraction suitable for COI barcode amplification while preserving shell integrity and fragile periostracal structures. High‐quality full‐length sequences were obtained from 76% of the samples, and 85% of the sequences were suitable for species identification. The remaining samples (15%) did not yield usable sequence data, possibly due to poor preservation, insufficient buffer access in tightly closed shells, or potential PCR problems. Specimens with fully closed opercula showed reduced success unless buffer access was facilitated. Our results show that HotSHOT is a fast and inexpensive method for DNA extraction that preserves shell vouchers, making it ideal for large‐scale barcoding of retracted microgastropods and valuable when working with rare specimens. By its low cost and high speed, it has a high potential to accelerate species discovery and delimitation, diversity assessments and monitoring and thus support conservation.

## Graphical abstract

Gastropods are among the most species‐rich animal groups, making rapid, voucher‐preserving DNA extraction methods essential for large‐scale genetic identification. We show that a nondestructive HotSHOT alkaline lysis protocol can recover COI sequences from ethanol‐preserved, retracted microgastropods while preserving shell integrity. The method is fast, inexpensive, and well suited for high‐throughput work and rare specimens.

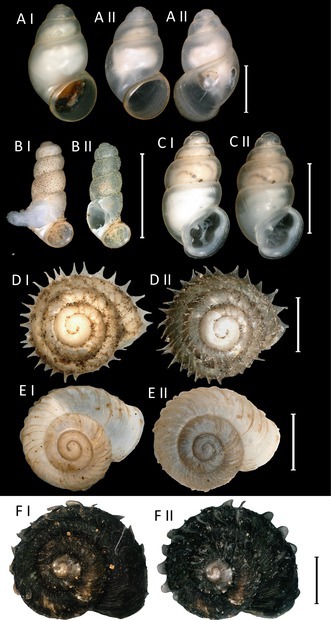

## 1 Introduction

Mollusks constitute the second most diverse animal phylum after arthropods, with approximately 100,000 to 170,000 described species (Ponder and Lindberg 2008; Strong et al. 2008). Among mollusks, gastropods represent the richest group, with estimates ranging from 65,000 to 120,000 described species (Strong et al. 2008; Appeltans et al. 2012; Rosenberg 2014; Rosenberg et al. 2022; Bánki et al. 2025), the current number of accepted extant species captured in the MolluscaBase is currently nearly 76,000 (MolluscaBase 2025), corresponding to 5% of the 1.5 million described animal species worldwide (Bánki et al. 2025).

A substantial portion of this diversity is comprised of microgastropods, typically measuring less than 5 mm, as well as other small‐sized gastropods. Data on size distribution are scarce, but for example, Bouchet et al. (2002) reported that over 50% of marine gastropod species from New Caledonia collected in their study were less than 8.8 mm in shell length, with 30% smaller than 4.1 mm. Similarly, a study of terrestrial gastropods of the Western Ghats in India by Aravind et al. (2008) found that of all gastropods found in that study, 40% were microgastropods. Taking a conservative estimate that one third of the 76,000 known gastropod species are microgastropods, this would correspond to approximately 25,000 species, representing about 1.7% of animal species worldwide.

Estimates on yet unknown or undescribed gastropod diversity suggest another 109,000–131,000 species (85,000–105,000 marine, (Appeltans et al. 2012; Rosenberg 2014), 4000–6000 freshwater (Strong et al. 2008), 20,000 terrestrial species (Rosenberg et al. 2022)), more than doubling the total number to roughly 200,000. Combined with the fact that most faunal surveys and species descriptions have historically been biased toward larger species (Bouchet et al. 2002; Strong et al. 2008), it seems reasonable to estimate the share of small and microgastropods to more than 35%–40%, thus roughly 70,000–80,000 species. Given both their vast numbers and the chronic under‐sampling of these tiny taxa, there is a clear need to develop suitable nondestructive, rapid, and cost‐effective molecular methods to enable basic research in this group. Because their small size makes species delimitation based on morphology challenging, incorporating molecular data is essential for detecting new, often morphologically cryptic, species (Weigand et al. 2012, 2013; Haase and Zielske 2015; Layton et al. 2019). Notably, this need is further underscored by the high conservation relevance of gastropods, which represent the animal class with the highest documented extinction rates, accounting for 37% of recorded invertebrate extinctions (Lydeard et al. 2004).

However, extracting DNA from preserved microgastropods retracted into the shell (with the animal's body fully withdrawn in its shell) poses a significant problem: Either the shell must be broken to remove the soft body or the whole animal with its shell will be exposed to extraction chemicals. Using established kits or protocols, the latter usually leads to severe damage of the calcareous shell and/or the organic outer layer, the periostracum. This is especially problematic when dealing with rare material or type specimens, where preserving the shell is essential for future morphological study.

While nondestructive DNA extraction techniques have been developed for various other organism groups, such as arthropods (Castalanelli et al. 2010; Porco et al. 2010; Miura et al. 2017) and plants (Shepherd 2017; Kelley et al. 2020), comparable methods for preserved gastropods remain limited. For gastropods, researchers extract DNA from entire gastropod specimens, mostly still breaking and removing the shell in the process (Weigand et al. 2013; Zielske and Haase 2015; Criscione et al. 2017; Khalik et al. 2019; Layton et al. 2019; Gyeltshen et al. 2023; Bullis and Rundell 2024).

There are some ways to avoid breaking the shell, such as the traditional flash‐boiling and subsequent removal of the body before preservation, more recently described as Niku‐nuki method (Fukuda et al. 2008). But this method is quite work‐intensive, requires equipment that is not always available under field conditions, and is not always feasible, for example for very small specimens or specimens with specific shell forms. Obvious limits are reached when the animal remains retracted or for fixed samples. Other approaches have extracted DNA from mollusk shells which can be suitable for larger mollusks (Martin et al. 2021; Goulding et al. 2021; Efstratiou et al. 2025). However, these techniques are not applicable to microgastropods for nondestructive DNA extraction, as the amount of shell available is insufficient; in the case of shell extraction, obtaining enough material would consume most or all of the shell, leaving no voucher specimen. There are some studies (Weigand 2013; Inäbnit et al. 2019, 2021) using the GeneReleaser method, developed for metazoans (Schizas et al. 1997) adapted by Böttger‐Schnack and Machida (2011) as a nondestructive method for gastropods. Nevertheless, the limited adoption of this method suggests it may not be optimal and own trials showed significant damage to the shells.

During a project on New Caledonian groundwater microgastropods of the family Tateidae with often very limited material, including undescribed species, we needed a less destructive and efficient DNA extraction method that would reliably allow at least single locus sequencing such as the partial COI gene. While searching for such a method, we turned our attention to protocols where the shell is in the lysis buffer as short as possible and at the same time exposed to buffers that are more alkaline. One particularly promising method was the HotSHOT (hot sodium hydroxide and Tris) (Truett et al. 2000) method with a lysis time of 30 min and a pH of 12 for the lysis buffer.

The HotSHOT method is widely used as a cost‐effective, fast, and easy DNA extraction for many different organism groups (Montero‐Pau et al. 2008; Labrador et al. 2019; García‐Abolafio et al. 2023). Several studies have demonstrated its use as a nondestructive method, particularly for insects (Alasaad et al. 2008; Guzmán‐Larralde et al. 2017; Suaste‐Dzul et al. 2019; Srivathsan et al. 2019; Fowler et al. 2023). While there are studies using this method for mollusk tissue clippings or larvae (Zieritz et al. 2018; Kartavtsev et al. 2018; Nguyen et al. 2021), it has yet to be tested as a shell‐preserving nondestructive method for mollusks inside their shells.

In this study, we assessed the HotSHOT extraction method for microgastropods that were fixed in a retracted state. We focused on two main aspects: the preservation of the shell and the success of single locus sequencing, here specifically targeting the DNA barcode fragment of the mitochondrial COI gene.

## 2 Materials and Methods

**FIGURE 1:**
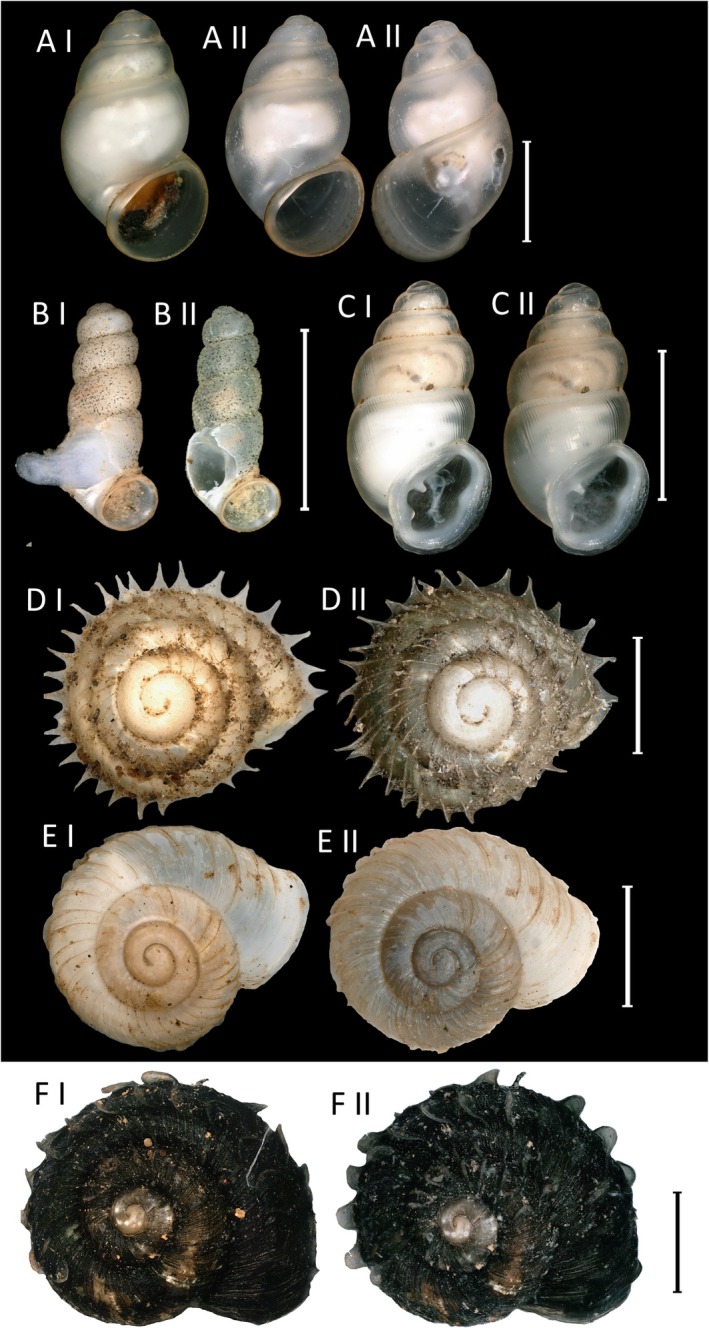
Comparative images of specimens before (I) and after (II) HotSHOT extraction. (A, B) *Tateidae* sp.; (C) *Carychium tridentatum* ; (D) *Acanthinula aculeata*; (E) *Vallonia costata* ; (F) Charopidae, *Acanthoptyx* sp. Scale bars = 1 mm.

A total of 109 specimens were used in this study with a size range from roughly below 1–5 mm, encompassing both operculate and non‐operculate species with varying shell structures including different periostracal features. All specimens were preserved in 96% ethanol. Ideally, operculate specimens were killed crawling by brief exposure to boiling water for a few seconds prior to preservation to prevent full retraction and tight sealing of the aperture. For almost all closed operculate specimens, the operculum was carefully removed when feasible, or alternatively, a small hole was created in the back of the shell (Figure 1, panel A) to allow lysis buffer penetration. For details about preparation, see the Table S1. To explore the amount of possible shell damage, the specimens were photographed before and after extraction using a Keyence VHX digital microscope.

### 2.1 DNA Extraction

The alkaline lysis and neutralizing buffers were prepared following Truett et al. (2000). After removing the specimens from ethanol, they were briefly dried on a paper tissue to allow the ethanol to evaporate and then placed in a tube (individuals below 2 mm in 0.2 mL PCR tubes and specimens above 3 mm in 1.5 mL tubes) and covered with alkaline lysis buffer (pH 12); for exact volumes, see Table S1. The specimens were fully submerged in the liquid before heating them to 95°C for 28 min, followed by 2 min at 98°C, and then cooled to 25°C (Vasilita et al. in press). Immediately after completing the alkaline lysis step, an equal volume of neutralizing buffer (pH 5) was added, followed by a brief vortex. The DNA extract was carefully transferred to a new tube. The remaining shell was rinsed with water to remove remaining buffer and transferred back to ethanol.

### 2.2 PCR

To assess success of the DNA extraction, the DNA barcode fragment of the cytochrome oxidase I (COI) marker was amplified and sequenced.

The PCR reactions were prepared in a total volume of 25 μL, with 1 μL of each primer LCO1490 and HCO2198 (Folmer et al. 1994) both at a concentration of 10 μM, 2.5 μL of dNTPs (10 mM), 0.2 μL of DreamTaq DNA Polymerase and 2.5 μL of 10X DreamTaq Buffer (Thermo Fisher Scientific), 1 μL of Bovine serum albumin (BSA) at 10 mg/mL, 1–3 μL of template DNA (see Table S1 for specific volume), and nuclease‐free water to the final volume. The PCR was carried out with the following protocol: an initial denaturation at 95°C for 5 min; followed by 35 cycles of 95°C for 30 s, 45°C for 45 s, and 72°C for 1 min; and a final extension at 72°C for 5 min.

The PCR product was checked via electrophoresis on a 1% agarose gel, followed by sequencing if a band (even a faint one) was visible. A total of 46 samples were sequenced by Sanger sequencing in both directions (LGC Biosearch Technologies, Berlin), while sequencing for 52 samples was performed in‐house through Nanopore sequencing with the MinION (the amplicons sequenced with the MinION were amplified with tagged primers following Srivathsan et al. 2019). Amplicon pooling, clean‐up, and library preparation followed the protocol described in Srivathsan et al. (2019).

### 2.3 Sequence Analyses

The sequences obtained by Sanger sequencing were assembled and edited using Geneious Prime 2023.2.1 (https://www.geneious.com). For Nanopore, basecalling was performed under the super accuracy model with Guppy v 6.4.2 + 97a7f0659. Demultiplexing and barcode calling were performed with ONTbarcoder v2.2 as described in Srivathsan et al. (2024). The sequences were then classified into three groups: (1) high‐quality sequences that were full length (658 or 655 base pairs); (2) partial sequences that were not full length due to unclear reads in one direction for Sanger sequencing or the presence of ambiguous bases (Ns) in nanopore sequences; and (3) failed sequences for which no usable data were obtained. For Sanger sequencing, sequences with occasional IUPAC ambiguity codes were not considered partial if they otherwise had high‐quality coverage. For Nanopore data, sequences were classified as partial if they contained a stretch of more than 30 ambiguous bases (N); for details and sample counts, see Table S1.

The sequences were then validated using the BLAST algorithm (Altschul et al. 1990) on the NCBI website (https://www.ncbi.nlm.nih.gov) or by aligning them with sequences from our own database in case of missing reference data.

## 3 Results

The here tested HotSHOT extraction method proved to be nondestructive, as all shells and their important morphological characters remained intact following treatment, including delicate or highly structured periostracal features (Figure 1, panel D, E and F), extremely thin shells (Figure 1, panel B), and fine structures such as ribs or apertural teeth (Figure 1, panel C). Notably, the soft body was often partially or completely dissolved during the extraction process, indicating effective tissue lysis without compromising shell integrity.

In operculate snails, when the snail was fully retracted into the shell and tightly sealed by a thick operculum, the soft body showed no signs of lysis, suggesting that the lysis buffer was unable to penetrate the shell. Much better buffer access and tissue degradation were observed in samples where penetration was made possible either by prior adequate treatment (e.g., pouring boiling water on the crawling specimen for a few seconds to prevent full retraction) or by removing the operculum or piercing the back of the shell for fully retracted specimens.

**FIGURE 2:**
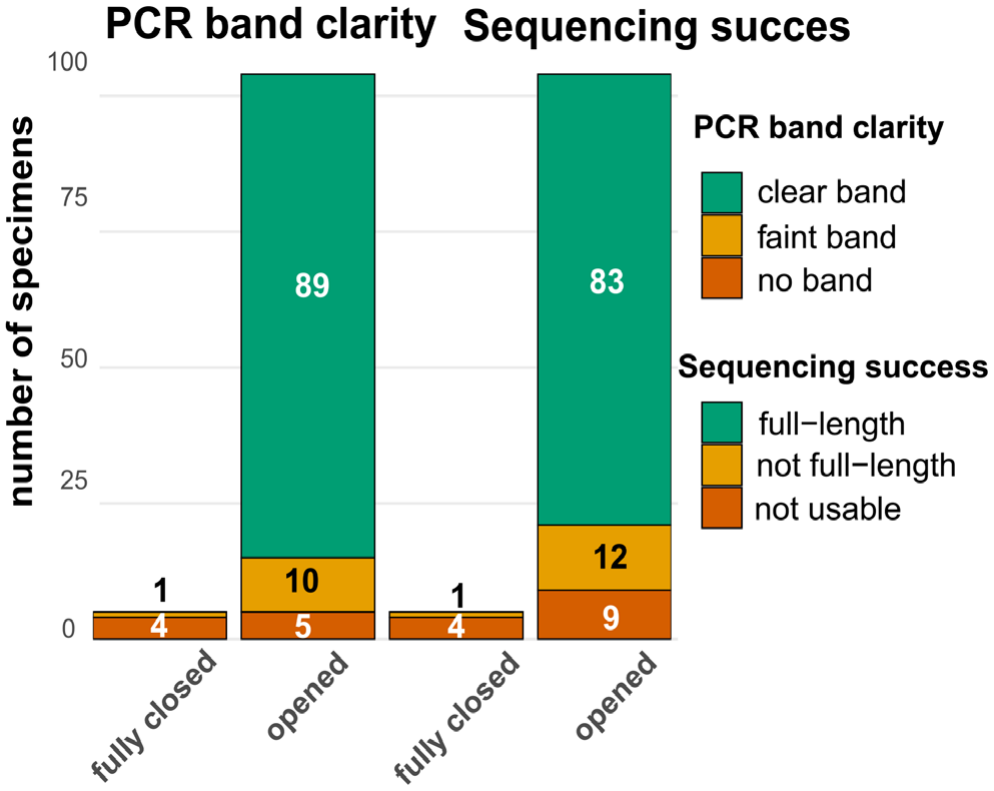
Barplot of PCR band clarity and sequencing success in specimens with fully closed versus opened shells.

This was also reflected in PCR success (Figure 2): Four of the five extracts from tightly closed operculate individuals failed to amplify, while only one showed a faint band, supporting the observation of poor lysis. In contrast, samples from partially open specimens (*n* = 104) had a lot higher amplification success rate. Of these 104 samples, 89 showed clear bands, 10 showed weak bands, and five failed to amplify. So, out of the total 109 samples, 89 had good bands, 11 weak, and 9 no bands at all.

Of the 89 samples with clear bands, high‐quality sequences were obtained for 79 samples; eight gave sequences that were not full length, and only two failed to generate any sequences.

The 11 samples with weak bands yielded four high‐quality sequences, while five (including the tightly sealed specimen) produced sequences that were not full length or had ambiguous sites and two failed to generate any useful sequences. In total, 83 samples yielded high‐quality, full‐length sequences; 13 produced shorter sequences, and 13 failed to generate any sequences. Of the failed samples, three were tightly closed, making lysis very difficult. When comparing sequences via BLAST or with our own reference sequences of the same taxa, all successfully sequenced samples, including those with shorter sequences, were correctly assigned to their respective taxa.

These results demonstrate that a majority of samples (76%) yielded high‐quality sequences, and an even greater proportion (85%) provided data sufficient for confident species‐level assignment.

## 4 Discussion

Compared with other DNA extraction methods for retracted snails, the shells showed no signs of destruction. This is probably due to the alkaline pH of the lysis buffer (pH 12) and the short extraction time. Notably, even the delicate and complex periostracum remained intact, demonstrating that this method is both gentle and effective in preserving the shell's fine details.

Our observed success rate (85%) for samples that yielded sequences suitable for single‐gene (COI) barcoding may still underestimate the true potential. Some specimens that were fully closed during fixation might not have been well preserved since the ethanol might not have penetrated the soft body. In these cases, DNA degradation could have occurred before extraction, limiting successful DNA isolation regardless of the lysis method. Preventing full closure at fixation can therefore improve preservation and extraction success. Therefore, the true DNA extraction success from well‐preserved specimens might be higher than reported, as poor fixation cannot be ruled out in some of our specimens.

The overall success rate could potentially be improved further through optimization of PCR conditions. For specimens from the Tateidae family of New Caledonia, obtaining reliable results was especially crucial. Therefore, PCR protocols were more extensively optimized, including increasing DNA template volumes, which sometimes enhanced amplification success. More generally, PCR success in some samples may have been limited by co‐extracted inhibitors such as salts, mucopolysaccharides, polyphenols, or other secondary metabolites naturally present in mollusks, which can persist in crude extracts and inhibit enzymatic reactions even when DNA extraction appears effective (Layton et al. 2014; McKee et al. 2015). While strategies like sample dilution can sometimes mitigate their impact, an additional column‐based purification step could further remove inhibitors, potentially improving PCR success rates and at the same time enhancing long‐term stability of DNA extracts. However, this would inevitably increase time and cost and thus compromise some of the main advantages of the HotSHOT protocol, but may be justified for particularly rare or irreplaceable material. Additionally, in cases where one sequencing direction produced a high‐quality read but the other did not, this likely indicates successful DNA extraction but suboptimal PCR amplification in one direction. This may be due to primer binding inefficiency rather than poor DNA quality. Since only the universal Folmer primers were used in this study, exploring alternative primers or PCR protocols might further improve amplification success and sequence quality, especially for taxa with variable primer binding sites.

In this study, 76% of the sequences were high‐quality and full length (655–658 bp), providing sufficient information for robust phylogenetic analyses. Importantly, shorter sequences, down to 520 bp, still enabled reliable species identification through BLAST searches or accurate placement in phylogenies. This aligns with previous research showing that much shorter fragments, known as mini‐barcodes, can perform comparably to full‐length barcodes when exceeding approximately 200 bp in length (Yeo et al. 2020). Since our shortest sequences are longer than these commonly accepted mini‐barcode sizes, this further supports the reliability of our data even when complete sequences are not obtained.

Furthermore, we confirm the other advantages of the HotSHOT protocol as already noted by other authors (Montero‐Pau et al. 2008; Meissner et al. 2013; Amezcua‐Martínez et al. 2025): The method is easy to implement, time efficient with an extraction time of slightly more than half an hour and is extremely low‐cost with extraction cost per sample at less than 0.025 € (Meeker et al. 2007) with buffers that can easily be mixed in the laboratory. Therefore, the HotSHOT method is also perfectly suitable for large scale studies, as already demonstrated for insects (Hartop et al. 2022).

While the HotSHOT approach has many advantages, it does carry some limitations compared to more elaborate extraction protocols. Because HotSHOT relies solely on alkaline lysis and neutralization without any column‐based or organic‐solvent purification, cellular debris, proteins, and salts remain in the lysate (Amezcua‐Martínez et al. 2025). These residual inhibitors can interfere with downstream enzymatic reactions, particularly if one attempts long‐range PCR or multiplex assays. Moreover, the combination of high pH and heat inherent to HotSHOT tends to fragment genomic DNA into relatively small pieces (often < 1–5 kb), making the extracts unsuitable for workflows that require high‐molecular‐weight DNA such as genome assembly or long‐range amplifications (Morono et al. 2014). However, a recent study shows that HotSHOT specimens can be successfully re‐extracted with methods dedicated for the recovery of HMW DNA (Feng et al. 2025).

Another consequence of the lack of clean‐up is reduced storage stability: while DNA extracted with column‐based methods can be stored at −20°C for years with little loss of integrity, alkaline‐lysis methods, containing residual base and EDTA, are best used immediately or within days. Prolonged storage can lead to further strand breakage and loss of amplifiable template, although it has also been shown that at least after 3 months the extractions still were good enough for PCR (Truett et al. 2000). To test this, we conducted a small experiment by using DNA extracts stored for 1 year frozen at −20°C and found that they still amplified successfully in PCR, suggesting longer‐term usability than generally expected.

Importantly, none of these drawbacks undermine our intended application. Since our goal was rapid single‐gene (COI) barcoding rather than the extraction of high‐molecular‐weight or ultra‐pure DNA, we deliberately prioritized shell preservation, speed, and cost over DNA yield and long‐term stability.

Taking these considerations into account, our approach is particularly well‐suited to address the challenges presented by the vast and largely unknown diversity of microgastropods worldwide. Our HotSHOT‐based approach offers a promising solution by enabling reliable DNA extraction suitable for single‐gene (COI) barcoding without damaging the shells. By facilitating basic genetic analysis of such small and often understudied taxa, this method could significantly accelerate species discovery and delimitation efforts, as well as diversity assessments and monitoring activities, and thus support conservation efforts.

## Author Contributions

**Nina Tombers:** conceptualization (equal), methodology (equal), visualization (equal), writing – original draft (equal), writing – review and editing (equal). **Cristina Vasilița:** conceptualization (equal), methodology (equal), resources (equal), writing – review and editing (equal). **Ira Richling:** conceptualization (equal), methodology (equal), resources (equal), supervision (equal), validation (equal), writing – original draft (equal), writing – review and editing (equal).

## Funding

The authors have nothing to report.

## Conflicts of Interest

The authors declare no conflicts of interest.

## Supporting information


**Table S1:** Summary of DNA extraction and sequencing success for samples. For each sample, the shell approximated size (mm), shell condition, total extraction buffer volume (μl), DNA template volume used in PCR (μl), PCR gel results, sequencing method, sequence quality, and sequence length (bp) are shown. Green shading indicates high‐quality sequencing results; orange shading highlights medium‐quality results. All samples showed PCR amplification unless otherwise indicated.

**Data S2:** Fasta files for medium‐quality samples.

## Data Availability

The vouchers of the processed specimens are deposited in the collections of the State Museum of Natural History Stuttgart (SMNS) and the Muséum national d'histoire naturelle, Paris (MNHN). All DNA extracts are stored at the SMNS. GenBank accession numbers (PZ584947–PZ585029) for the high‐quality sequences are provided in Table S1. Medium‐quality sequences are available in Data S2.
